# A case report of sevelamer-associated recto-sigmoid ulcers

**DOI:** 10.1186/s12876-016-0441-4

**Published:** 2016-02-24

**Authors:** Christina Tieu, Roger K. Moreira, Louis M. Wong Kee Song, Shounak Majumder, Konstantinos A. Papadakis, Marie C. Hogan

**Affiliations:** Department of Internal Medicine, Mayo Clinic, Rochester, MN 55905 USA; Department of Anatomic Pathology, Mayo Clinic, Rochester, MN 55905 USA; Division of Gastroenterology and Hepatology, Department of Medicine, Mayo Clinic, Rochester, MN 55905 USA; Division of Nephrology & Hypertension, Department of Medicine, Mayo Clinic, 200 First Street SW, Rochester, MN 55905 USA

**Keywords:** End-stage renal disease, Hyperphosphatemia, Phosphate binder, Recto-sigmoid ulcers, Rectosigmoiditis, Sevelamer

## Abstract

**Background:**

Optimal phosphorous control is an important aspect of the care of patients with end-stage renal disease, and phosphate binders are usually needed.

**Case presentation:**

A 74-year-old woman with end-stage renal disease on maintenance hemodialysis presented to the emergency room with abdominal discomfort, rectal pain, and blood-tinged stools. Initial concern was for a rectal carcinoma, based on the symptoms and imaging in initial computerized tomography of the abdomen showing rectal wall thickening, and her clinical presentation. She had been treated with the phosphate binder sevelamer for two months. In this case report, we explore the unique features of sevelamer-associated recto-sigmoid ulcers which led to her symptoms.

**Conclusion:**

Sevelamer is widely used in chronic kidney disease and end-stage renal disease patients with hyperphosphatemia. It is a crosslinked polymeric amine that binds phosphates and bile acids; it is not systemically absorbed. To the authors’ knowledge, this is the first reported case of recto-sigmoid ulcers associated with use of this phosphate binder. Nephrologists, pathologists, and gastroenterology sub-specialists should be aware of this recently-reported entity in patients on sevelamer with suggestive symptoms, as this medication is widely used in renal patients.

## Background

Hyperphosphatemia is one of the most frequent metabolic derangements seen in end-stage renal disease (ESRD). Large observational studies have identified hyperphosphatemia as an independent risk factor for cardiovascular disease and mortality for patients on dialysis, and subsequent studies have found that subtle increases in serum phosphate levels within the normal range are also associated with increased risk for death in predialysis and even non–kidney disease populations [1–3]. Current practice guidelines recommend more aggressive management of hyperphosphatemia to lower serum phosphate targets than in the past [1, 4, 5]. Simultaneously, there has been an increase in utilization of non-calcium phosphate binders and several new resin-based products that have been approved by the Food and Drug Administration (FDA) for this indication, including sevelamer hydrochloride, a non-absorbed synthetic polymer [6–9]. *Renvela* (sevelamer carbonate) was FDA-approved in March 2008 and was preceded by sevelamer hydrochloride [9]. The market represents an estimated $1 billion for this drug alone in worldwide sales [10]. Various gastrointestinal side effects have been reported including vomiting (22 %), nausea (20 %), diarrhea (19 %), dyspepsia (16 %), abdominal pain (9 %), constipation (8 %) and associated stercoral ulceration, flatulence (8 %), intestinal obstruction, and fecal impaction [11–14]. However, there is little information regarding gastrointestinal ulceration as a side effect; only a report of sevelamer crystals isolated from the gastrointestinal tracts of patients with symptoms of gastrointestinal distress have been identified in the literature [15].

## Case presentation

**Fig 1:**
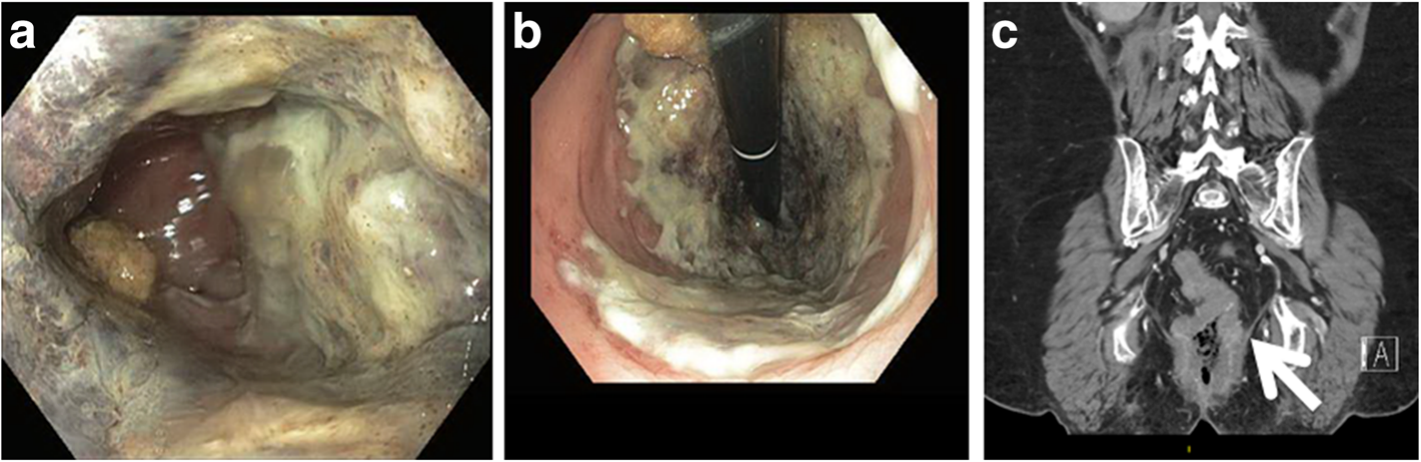
**a** and **b** Rectal mucosa with ulcerations, exudates, and purplish hue. **c** CT scan showing circumferential rectal wall thickening (arrow) with adjacent fat stranding and apparent adhesion to the mesorectal fascia

A 74-year-old woman with ESRD secondary to diabetes and maintained on hemodialysis three times per week presented to the Emergency Department with constipation, lower abdominal discomfort, prominent rectal pain, and blood-tinged stools. She had no watery diarrhea. Physical exam was significant for diffuse abdominal tenderness. Initial work up was significant for stable complete blood count and basic metabolic panel, with a stool specimen positive for *C. difficile* toxin by PCR in the context of recently treated *C. difficile* infection. Notably, symptoms were different from her prior episode of *C. difficile* associated diarrhea 11 months earlier. This was felt to be representative of a prior infection. Computed tomography (CT) of the abdomen and pelvis revealed non-specific circumferential rectal thickening with fat stranding suggestive of an inflammatory/infectious or malignant process (Fig. 1).

Sigmoidoscopy with biopsies was performed to establish a diagnosis, which revealed circumferential ulcerations, exudates, and purplish hue to the mucosa in the mid- and distal rectum consistent with ischemia. No classic endoscopic findings consistent with *C. difficile* infection, such as pseudomembranes, were identified.

**Fig 2:**
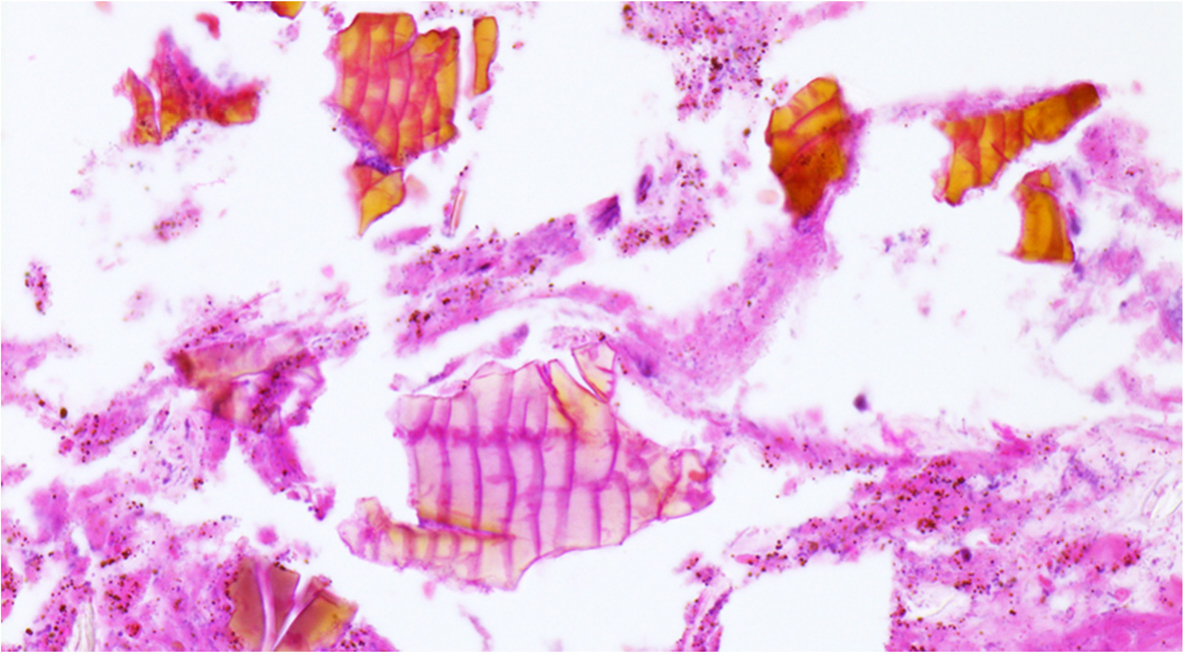
Characteristic histologic appearance of sevelamer pill fragments, featuring crystalloid structures with broad, slightly-curved, irregular “fish scales” with a two-toned (pink and tan-yellow) color seen on mucosal biopsy. (Hematoxylin and eosin stain, 400x magnification)

Endoscopic biopsies of the rectal ulcerations revealed pill fragments consistent with sevelamer crystals in the exudate. Sevelamer crystals in the fibropurulent/ necrotic debris identified on histology displayed a characteristic “fish scale” pattern [2] as shown in Fig. 2. No crystals were present within the preserved tissue fragments in the sample. Overall the findings were that of an ulcer without specific features to point to any other particular etiology (e.g. inflammatory bowel disease, ischemia etc.). The patient had commenced sevelamer (*Renvela* 1,600 mg orally 3 times a day) approximately 2 months prior to her presentation. Sevelamer-induced mucosal injury was confirmed, the medication was discontinued, and treatment with calcium acetate was substituted. Her constipation resolved with conservative medical management. Her incidentally positive *C. difficile* PCR was not felt to represent true infection, but was conservatively treated with a 10-day course of oral vancomycin.

## Discussion

Sevelamer-associated gastrointestinal mucosal injury is a relatively novel entity among sevelamer users that has not yet been fully characterized. In addition, its non-specific presentation on both CT imaging and endoscopy may lead to delayed recognition and under-diagnosis. Therefore, physicians should have a high index of suspicion to pursue further evaluation.

Unfortunately, an understanding of the incidence of sevelamer-associated gastrointestinal ulceration is limited by the sparse literature that is currently available. Reported sites of gastrointestinal mucosal involvement include the esophagus (*n* = 2), small bowel (*n* = 2), and colon (*n* = 12; including this case). It is currently unclear whether sevelamer was the causative agent of mucosal injury, or whether the association was coincidental in ESRD. However, we do not believe that sevelamer was incidentally adherent to the mucosa as a result of an underlying colonic disease process, including IBD, CMV colitis or diverticulitis, since no such intestinal pathology was documented on the biopsies. We, therefore, favor that sevelamer was the primary cause for the rectal ulcers. While we would have liked to confirm resolution of mucosal involvement following discontinuation of sevelamer to be 100 % confident of causality, this was not feasible due to burden of care relating to complications of kidney disease (access surgery, dialysis visits) lack of eligibility of kidney transplant and other comorbidities (endocarditis, eye surgery, diabetes care visits, hospitalization for cerebral infarction) that limit her life span with additional endoscopy being unlikely to improve quality of life or survival [16].

## Conclusions

We report the first clinical description of sevelamer induced rectal ulcers in a hemodialysis patient presenting with abdominal and rectal pain, constipation, and a mass on CT. Initial concern in this case was for a rectal carcinoma, which was ruled out with rectal mucosal biopsies showing characteristic sevelamer crystals. Furthermore, the mechanism by which sevelamer may have induced mucosal injury is unknown. In patients taking sevelamer, nephrologists, gastroenterologists and pathologists should be aware of the drug’s characteristic morphology to permit accurate diagnosis on biopsy and to reliably distinguish it from other gastrointestinal conditions.

### Consent

Informed consent was obtained from the patient for publication of this Case Report and any accompanying images.

## Abbreviations

CT: computed tomography
ESRD: end-stage renal disease
FDA: Food and Drug Administration
PCR: polymerase chain reaction
